# Voluntary exercise is motivated by ghrelin, possibly related to the central reward circuit

**DOI:** 10.1530/JOE-19-0213

**Published:** 2019-10-08

**Authors:** Hiroharu Mifune, Yuji Tajiri, Yusuke Sakai, Yukie Kawahara, Kento Hara, Takahiro Sato, Yoshihiro Nishi, Akinori Nishi, Ryouichi Mitsuzono, Tatsuyuki Kakuma, Masayasu Kojima

**Affiliations:** 1 Institute of Animal Experimentation, Kurume University School of Medicine, Kurume, Japan; 2 Division of Endocrinology and Metabolism, Kurume University School of Medicine, Kurume, Japan; 3 Department of Pharmacology, Kurume University School of Medicine, Kurume, Japan; 4 Molecular Genetics, Life Science Institute, Kurume University, Kurume, Japan; 5 Department of Physiology, Kurume University School of Medicine, Kurume, Japan; 6 Department of Exercise Physiology, Institute of Health and Sports Science, Kurume University, Kurume, Japan; 7 Bostatistics Center, Kurume University, Kurume, Japan

**Keywords:** exercise, ghrelin, motivation, reward circuit, dopamine

## Abstract

We previously reported that voluntary exercise contributed to the amelioration of abnormal feeding behavior with a concomitant restoration of ghrelin production in a rat model of obesity, suggesting a possible relationship between exercise and appetite-regulating hormones. Ghrelin is known to be involved in the brain reward circuits via dopamine neurons related to motivational properties. We investigated the relevance of ghrelin as an initiator of voluntary exercise as well as feeding behavior. The plasma ghrelin concentration fluctuates throughout the day with its peak at the beginning of the dark period in the wild-type (WT) mice with voluntary exercise. Although predominant increases in wheel running activity were observed accordant to the peak of plasma ghrelin concentration in the WT mice, those were severely attenuated in the ghrelin-knockout (GKO) mice under either *ad libitum* or time-restricted feeding. A single injection of ghrelin receptor agonist brought about and reproduced a marked enhancement of wheel running activity, in contrast to no effect by the continuous administration of the same drug. Brain dopamine levels (DAs) were enhanced after food consumption in the WT mice under voluntary exercise. Although the acceleration of DAs were apparently blunted in the GKO mice, they were dramatically revived after the administration of ghrelin receptor agonist, suggesting the relevance of ghrelin in the reward circuit under voluntary exercise. These findings emphasize that the surge of ghrelin plays a crucial role in the formation of motivation for the initiation of voluntary exercise possibly related to the central dopamine system.

## Introduction

It is well known that exercise in itself has several benefits in terms of health and fitness (Patterson & Levin 2008, Haskell-Luevano *et al.* 2009), as well as neural and cognitive effects (Cotman & Engesser-Cesar 2002), in both humans and laboratory animals. Food restriction and regular exercise are the two major established strategies for the prevention and treatment of obesity, which is currently recognized as a serious burden throughout the world. However, obesity is often associated with physical inactivity and disrupted life rhythms, including binge and night eating (Marcus & Wildes 2014), which makes the treatment of obesity more complicated and weight reduction less attainable. Although exercise is recommended for the purpose of weight reduction through the increment of energy expenditure, it is generally difficult for most obese subjects to continue regular exercise for long periods of time. Thus, it is important to explore the putative mechanisms for producing the motivation to perform and adhere to exercise, especially in obese subjects.

Ghrelin, which was originally identified as a growth hormone secretagogue (GHS), is an orexigenic gut hormone. This 28-amino acid peptide is produced by the X⁄A-like endocrine cells in the oxyntic glands of the gastric fundus (Kojima *et al.* 1999, Date *et al.* 2000). Ghrelin primarily functions as an orexigen (Nakazato *et al.* 2001) and as a GH-releasing hormone (Kojima *et al.* 1999). Furthermore, various other physiological roles have been reported, including the modulation of the energy metabolism (De Vriese *et al.* 2010), and the regulation of the autonomic nervous system (Matsumura *et al.* 2002, Date *et al.* 2006) and cardiovascular system (Tesauro *et al.* 2010). The biological activities of ghrelin require the octanoylation of the peptide on Ser_3_, an unusual post-translational modification that is catalyzed by the enzyme, ghrelin O-acyltransferase (GOAT) (Gutierrez *et al.* 2008, Yang *et al.* 2008).

Some earlier studies reported that in the absence of food, the administration of ghrelin increases locomotor activity and the subsequent intake of food, known as food anticipatory activity (FAA). In the absence of ghrelin receptors, this food anticipatory behavior is diminished (LeSauter *et al.* 2009), suggesting a crucial role of ghrelin in the formation of FAA. Thus, it has been hypothesized that ghrelin participates in the homeostasis of energy metabolism and the rhythm of daily activity, including eating behavior and locomotor activity. To provide an adequate energy balance, the food intake is regulated by a rewarding force as well as the sensation of hunger through the activation of other brain regions, such as the cerebral cortex and nucleus accumbens (NAc) (Phillips *et al.* 2007). Recent studies have shown that mesolimbic dopamine neurons in the ventral tegmental area (VTA) that project to the NAc represent a critical site for ghrelin to trigger food consumption behavior, since high levels of GHSR expression are recognized in the dopamine neurons in the VTA (Zigman *et al.* 2006). It is suggested that ghrelin is involved in the brain reward circuits that are related to motivational properties, as well as hedonic feeding.

We previously reported that voluntary exercise contributed to the amelioration of abnormal feeding behavior with a concomitant restoration of the blunted ghrelin production in a rat model of high-fat diet (HFD)-induced obesity (Mifune *et al.* 2015). Furthermore, it has been reported that endogenous ghrelin system is essential for exercise endurance by stimulating the sympathoadrenal system, increasing IGF-1 levels, and/or increasing the availability of gluconeogenic substrates such as lactate to meet the energy demand of prolonged exercise (Mani *et al.* 2018). It is thus plausible that a putative relationship may exist between exercise and this appetite-regulating hormone. As mentioned above, because ghrelin is relevant to higher motivation and hyperactivity (Blum *et al.* 2009, Menzies *et al.* 2013), we hypothesized that it plays an essential role as an initiator of voluntary exercise as well as feeding behavior. The present study was performed to clarify and validate the relevance of ghrelin in the motivation to perform voluntary exercise using ghrelin-knockout (GKO) mice.

## Materials and methods

### Animal

All animals were housed in a controlled room (temperature 25 ± 2°C, humidity 60 ± 10%) under a 12-h light-dark cycle (light on 07:00–19:00 h) with *ad libitum* or time-restricted feeding access to standard powder chow (10 kcal% fat, produced by Research Diets, Inc.: open source diet code D12450B) and water. All the experiments were performed in accordance with protocols approved by the Kurume University Animal Experiment Committee, based on the NIH Guidelines for the Care and Use of Laboratory Animals (NIH publication, 1996).

Ghrelin-knockout (*Ghrl^−/−^*) mice were produced as described previously (Sato *et al.* 2008). Mice with the same genetic background were generated by homologous recombination. Briefly, the coding region of the *Ghrl* locus (from ATG initiation codon to the termination codon) was deleted precisely from bacterial artificial chromosome (BAC)-based targeting vectors and replaced with a neomycin-selectable marker before electroporation into embryonic stem (ES) cells. The presence of the construct in correctly targeted ES cells and derived wild-type (*Ghrl^+/+^*), heterozygous (*Ghrl^+/−^*), and homozygous (*Ghrl^−/−^*) pups was identified by PCR. After establishing germ-line transmission, mice were backcrossed to wild-type C57BL6/J mice to generate N10 breeding heterozygote pairs. These pairs were used to generate homozygous null mice.

### Experimental protocols under *ad libitum* feeding

Male (wild-type) WT and GKO mice at 11 weeks of age were individually housed in ordinary clear plastic cages (W22 × D32 × H13 cm) with paper bedding before experiments.

After 1-week acclimatization period, mice at 13 weeks of age in exercise groups (WT-Ex and GKO-Ex; *n* = 8, respectively) were individually housed in specially designed PVC chambers (W32 × D20.5 × H26.5 cm) equipped with a running wheel apparatus (15 cm diameter and 5 cm width) (Supplementary Fig. 1, see section on supplementary materials given at the end of this article). Food intake and locomotor activity in the sedentary room were recorded automatically using a monitoring system (ACTIMO-1M combined with MFD-100-CH; Shinfactory, Fukuoka, Japan) in the individual chambers from 13 to 14 weeks of age. To measure locomotor movement, sensors are located every 2 cm along the floor of the enclosure (5 cm above the wire floor), and movement is detected by an infrared beam every 0.5 s. To eliminate any artifacts elicited by respiration or nose/tail movements, the simultaneous interruption of more than two neighboring beams are recorded as ‘an activity output’ by ACTIMO-SII software (Shinfactory, Fukuoka, Japan). Movement signal counts were imported using the Spike2 analysis program (Cambridge Electronic Design, Cambridge, UK). At the opposite end of the chamber there was a food intake monitor filled with standard powder chow as mentioned above. Food intake was recorded simultaneously every 3 min using a high accuracy scale that weighed the chow, and the minimum quantity measurable was 0.01 g of chow.

Wheel running activity was recorded automatically using a monitoring system (RW-RQ combined with MFD-100-CH; Shinfactory) in the exercise room of the chamber with which a running wheel is equipped to measure cumulative wheel running counts (Supplementary Fig. 1). All the wheel revolution counters were recorded as ‘wheel-running activity’ by ACTIMO software (Shinfactory) on a minute-by-minute basis (number of wheel revolutions).

All measurement data were recorded for 2 weeks, and data for the last 2 days of the experimental period were retrieved and analyzed.

### Experimental protocols under time-restricted feeding

Under time-restricted feeding, it is well known that an increase in locomotor activity and voluntary exercise occurs as an exploratory behavior (Hatori *et al.* 2012). Furthermore, if access to food is limited to a few hours a day, mice display increases in locomotor activity prior to the time of food presentation as FAA (Mistlberger 1994). To explore a potential effect of ghrelin on voluntary exercise or FAA under time-restricted feeding, another series of experiments were performed as mentioned below.

After 1-week acclimatization period, WT and GKO mice at 13 weeks of age were individually housed in the same chambers as above, and after 24-h fasting the restricted feeding (RF) protocol was performed for the next 2 weeks (WT-Ex-RF and GKO-Ex-RF; *n* = 6, respectively). Under RF condition, they were fed twice a day only between *Zeitgeber* time (ZT) 12 and 14, and ZT 22 and 24, in which ZT 0 is the time of lights on (4-h time-restricted feeding during the dark cycle, Supplementary Fig. 4). Food intake, locomotor activity and wheel-running activity were recorded for 2 weeks, and data for the last 2 days were retrieved and analyzed as mentioned above. Using this system, the RF is programmed and automatically performed so that mice are given access to standard powder chow only while the shutter device on feeder box is open twice a day (Supplementary Fig. 1).

### Administration of ghrelin receptor agonist

GHRP-6 (Bachem, Bubendorf, Switzerland) was used as a ghrelin receptor agonist. For once-daily administration group, GKO mice at 13 weeks of age were injected intraperitoneally with GHRP-6 (1 μg/g body weight, calculated as per day based on the dose of continuous administration below mentioned; *n* = 6) or vehicle (saline; *n* = 6) at 18:30 h for 14 days (from 13 to 14 weeks of age), and wheel-running activities were recorded throughout the period. For continuous administration group, the osmotic mini-pump (Alzet Model 1004; Duret Corporation, Cupertino, CA, USA) containing either saline (vehicle; *n* = 6) or GHRP-6 in saline (42 ng/0.11 μL/h/g body weight, known to promote food intake; Sato *et al.* 2011; *n* = 6) was implanted intraperitoneally under anesthesia in each GKO mouse at 12 weeks of age. After 1-week habituated period, wheel-running activities were recorded same as once-daily administration group from 13 to 14 weeks of age. Both measurement data were recorded for 2 weeks, and data for the last 2 days of experimental period were retrieved and analyzed.

### Surgery and brain dialysis for the measurement of dopaminergic activity

In WT-Ex and GKO-Ex mice, microdialysis was performed with an I-shaped cannula. A microdialysis probe was implanted in the unilateral NAc region (exposed length 1.5 mm) at 15 weeks of age (Supplementary Fig. 2) after voluntary exercise for 2 weeks (from 13 to 14 weeks of age). Surgery was conducted under pentobarbital anesthesia (50 mg/kg i.p.) and local application of 10% lidocaine. The stereotaxic coordinate of the implantation of the probes was A/P 1.4 mm, L/M 0.6 mm, V/D 4.5 mm from the bregma and dura for the NAc region. After surgery, the mice were housed individually in acrylic boxes (30 × 30 × 40 cm) and allowed to recover for 48 h before performance of microdialysis experiments as previously described (Kawahara *et al.* 2009, 2013, Kaneko *et al.* 2016). The recovery time of 48 h was adopted based on the previous report that the micordialysis experiments must be performed within an optimal time window, usually 24–48 h after the probe implantation (Di Chiara *et al.* 1996).

After 24 h of food deprivation, mice implanted a microdialysis probe were divided into three groups: WT-Ex, GKO-Ex, and GKO-Ex administrated with GHRP-6 (Bachem, Bubendorf, Switzerland) as a ghrelin receptor agonist which were injected intraperitoneally 1 μg/g body weight at 10 min before feeding (*n* = 5, respectively). Mice in each group were fed regular food for 20 min period, thereafter food was removed. Food amounts consumed for 20 min were comparable among WT-Ex (114.4 ± 65.6 mg), GKO-Ex (113.2 ± 38.1 mg) and GKO-Ex administrated with GHRP-6 (118.2 ± 40.8 mg). Dialysate fractions were collected before feeding (basal) and every 15 min after the beginning of food consumption. Dopamine level (DA) was quantified by HPLC with electrochemical detection conducted in the same manner as our previous study (Kawahara *et al.* 2013). At the end of the experiments, the mice were given an overdose of sevoflurane and brains were fixed with 4% paraformaldehyde via intra-cardiac infusion. Sagittal sections (50 µm) were cut and dialysis probe placement was confirmed using the atlas of Paxinos & Franklin (2004). Mice, in which dialysis probes were misplaced, were excluded from the data analysis. In the current experiment, the probe trace was not found clearly in one mouse in GKO-Ex with GHRP-6 group and the data from this mouse were excluded. Then, an additional experiment was performed in this group of mouse with the correct placement of the probe confirmed. Therefore, *n* = 5 is the final number of mice used for the analysis.

### Radioimmunoassay (RIA) for ghrelin

Male WT-Ex mice at 15 weeks of age after measurements for food intake, locomotor and wheel running activity, and age-matched male WT mice without exercise used as controls were killed under anesthesia with 3% isoflurane and blood samples were collected at 4 time points (07:00, 13:00, 19:00, 01:00 h) for the measurement of plasma active ghrelin concentrations by RIA. Blood samples were collected via abdominal aorta into pre-chilled EDTA-2Na tubes with aprotinin.

Plasma samples for ghrelin were prepared by adding hydrochloric acid (final concentration of 0.1 N) followed by extraction using a Sep-Pak C18 cartridge (Waters) (Kojima *et al.* 1999, Hosoda *et al.* 2000). The eluate was lyophilized and stored at −80°C until the assay was performed. Lyophilized samples were dissolved in RIA buffer on the day of assay. Active ghrelin RIAs were performed as previously described (Nishi *et al.* 2005*a*,*b*) using rabbit antisera against the N-terminal (Gly^1^-Lys^11^ with O-n-octanoylation at Ser^3^) fragments of rat ghrelin. This anti-rat ghrelin (1–11) antiserum, which specifically recognized the Ser^3^ n-octanoylated form of ghrelin, does not recognize des-acyl ghrelin, but cross-reacts faintly with other acyl-modified ghrelins, and exhibited complete cross-reactivity with human, mouse, and rat ghrelins.

### Statistics

**Figure 1:**
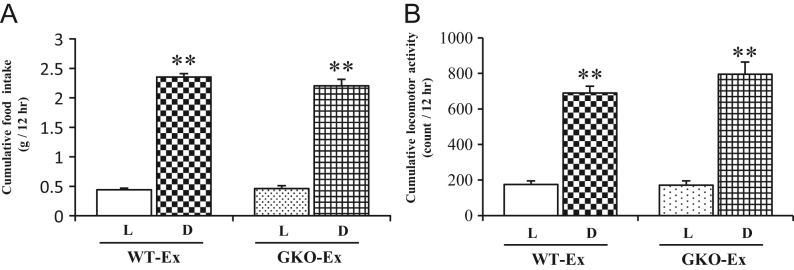
The food intake, locomotor activity and wheel-running activity under *ad libitum* feeding. The cumulative food intake (A) and locomotor activity (B) during the light and the dark periods were shown in either WT- or GKO-Ex mice. Values are means ± s.e.m. WT, *n* = 8; GKO, *n* = 8. L, light period; D, dark period. **P < 0.01 versus light period in each genotype of mice.

**Figure 2:**
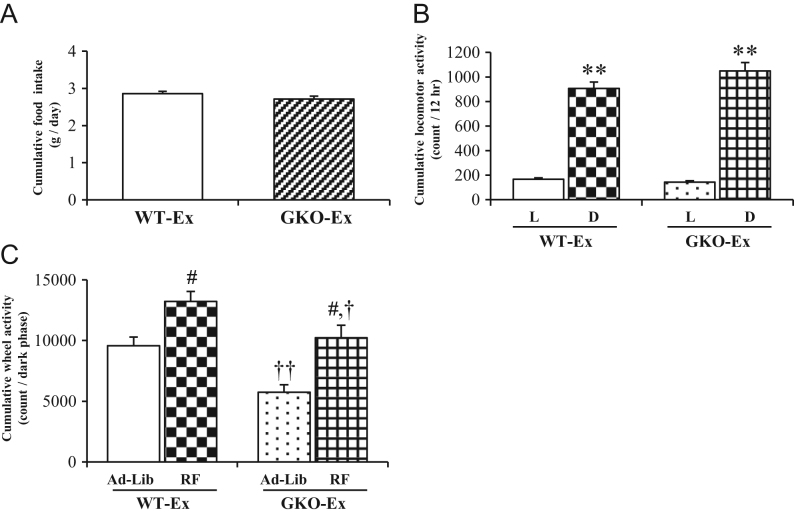
The food intake, locomotor activity and wheel-running activity under time-restricted feeding. The daily food intake (A) and locomotor activity (B) during the light and the dark periods were shown in either WT- or GKO-Ex mice. The cumulative wheel-running activity during dark period under restricted feeding was shown along with that under *ad libitum* feeding in either WT- or GKO-Ex mice (C). Values are means ± s.e.m. WT, *n* = 8; GKO, *n* = 8. L, light period; D, dark period, Ad-Lib, *ad libitum* feeding; RF, time-restricted feeding. **P < 0.01 versus light period in each genotype of mice. ^#^*P* < 0.01 versus *ad libitum* feeding in each genotype of mice. ^†^*P* < 0.05 versus WT-Ex-RF, ^††^*P* < 0.01 versus WT-Ex-Ad-Lib.

**Figure 3:**
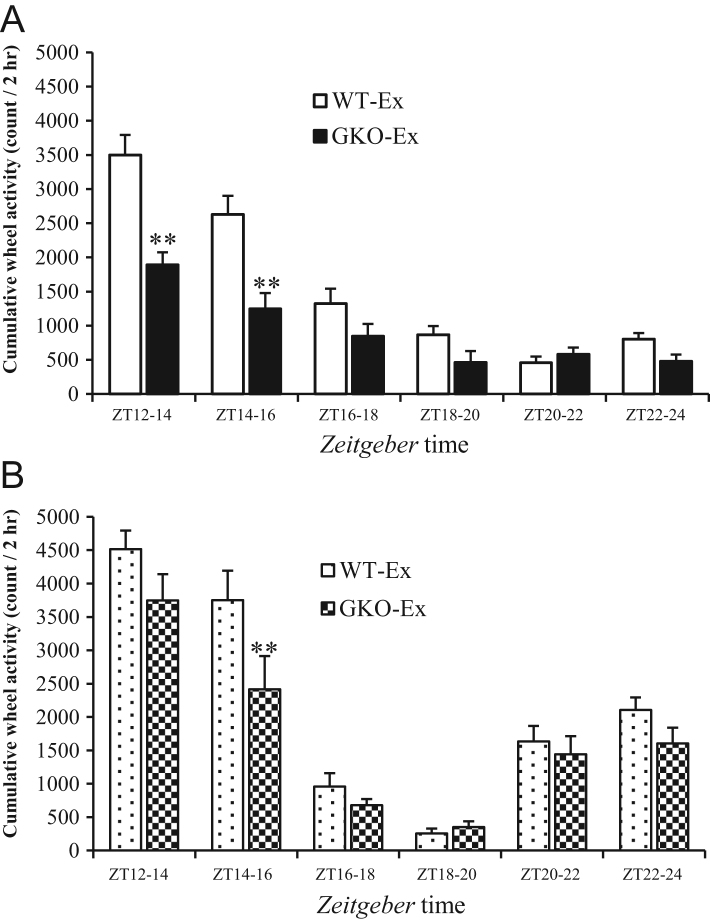
The patterns of wheel-running activity. The wheel-running activity of WT-Ex and GKO-Ex mice for every 2 h during the dark period under *ad libitum* feeding (A) or time-restricted feeding (B). Values are means ± s.e.m. WT, *n* = 8; GKO, *n* = 8, **P < 0.01 versus WT-Ex.

**Figure 4:**
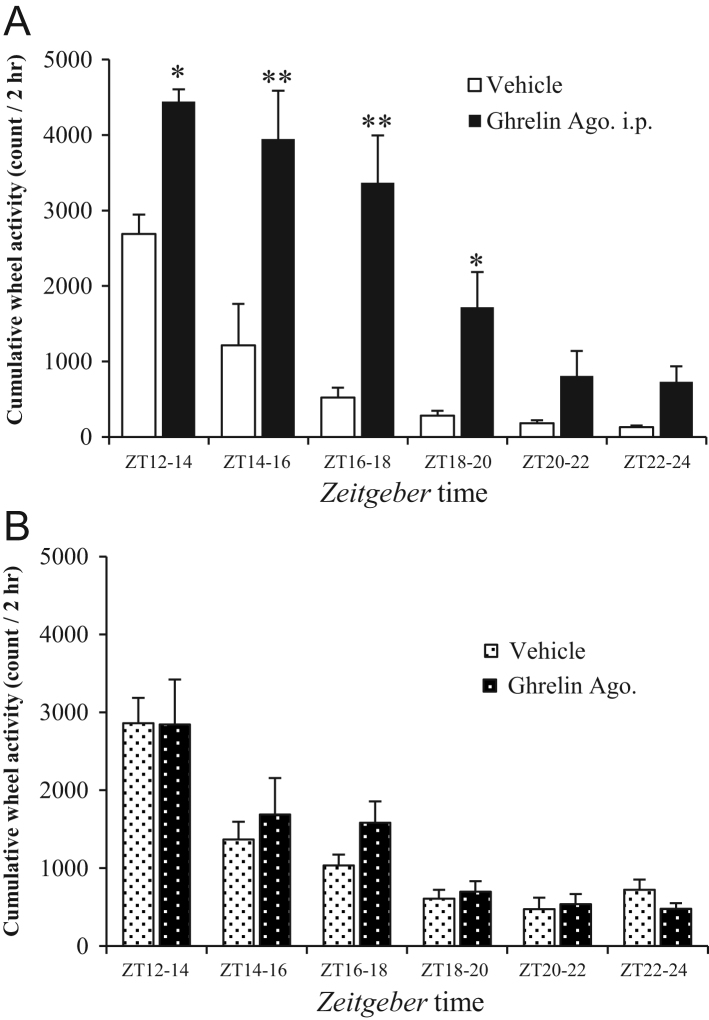
The effect of a ghrelin receptor agonist on voluntary wheel running activity in GKO mice. After the once-daily administration of GHRP-6 or vehicle at ZT 11:30 for 14 days, the wheel-running activity was recorded for every 2 h during the dark period (A). After the continuous administration of GHRP-6 by an osmotic mini-pump for 14 days, the wheel-running activity was recorded in the same way (B). Values are means ± s.e.m Vehicle, *n* = 6; Ghrelin Ago, *n* = 6. *P < 0.05 and **P < 0.01 versus vehicle at the same time point. Ghrelin Ago: ghrelin receptor agonist, ip: intraperitoneal injection.

**Figure 5:**
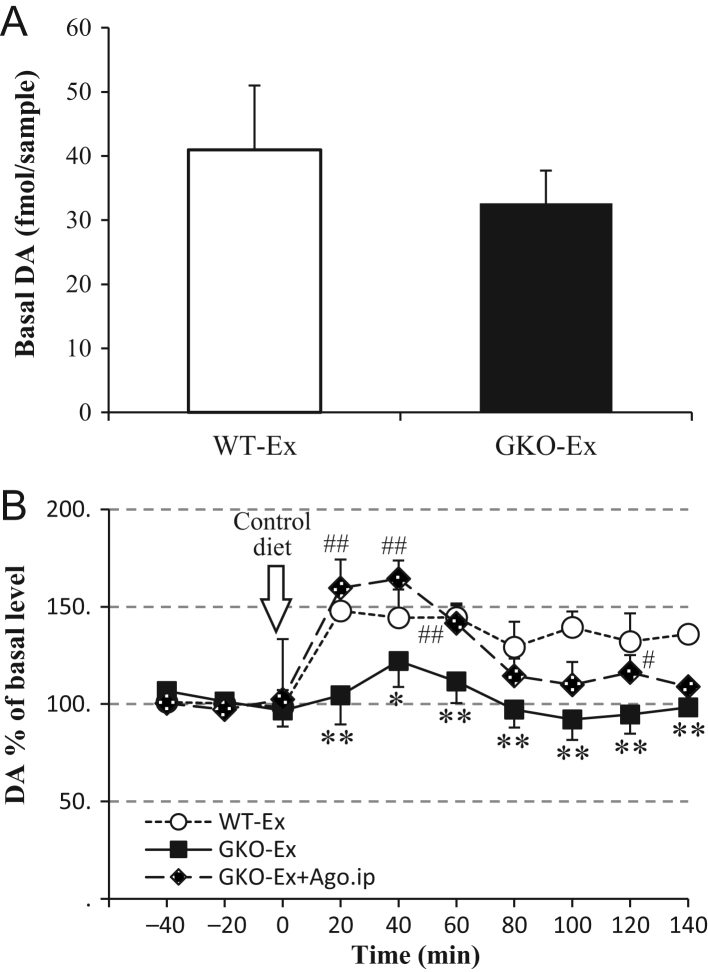
The effect of voluntary exercise for two weeks on the dopamine levels in the unilateral NAc. The basal dopamine (DA) levels in the NAc of WT-Ex and GKO-Ex mice (A) and the food consumption-induced increase in the DA levels in the NAc of WT-Ex, GKO-Ex and GKO-Ex administrated by GHRP-6 mice (B) were shown. Values are means ± s.e.m WT-Ex, *n* = 5; GKO-Ex, *n* = 5; GKO-Ex + GHRP-6, *n* = 5. *P < 0.05 and **P < 0.01 versus WT-Ex at the same time point. ^# ^*P *< 0.05 and ^##^*P* < 0.01 versus GKO-Ex at the same time point. Arrow indicates the time point at which the mice in each group began to be fed regular food for a period of 20 min after 24 h of food deprivation.

All tests were performed using SPSS Statistics Ver. 23 (IBM Corp.). Two- sample *t*-test was used for the comparisons between data in WT and GKO. To compare the effects of food restriction and genotype on wheel running activity simultaneously, two-way factorial ANOVA with Scheffe type multiple comparison test was performed (Fig. 2C). Mixed ANOVA was performed for the comparisons of food intake and locomotor activity between light and dark period concomitant with those between WT-Ex and GKO-Ex under* ad libitum* (Fig. 1A and B) or time-restricted (Fig. 2B) feeding. Mixed ANOVA was also performed for the comparison of FAA between ZT11-12 and ZT21-22 in each Ex-RF group and FAA between both genotypes simultaneously (Supplementary Fig. 5). Comparisons of chronological data between two groups of mice were performed using mixed model analysis (Figs 3, 4, 5B and Supplementary Fig. 6). The time-dependent change of plasma ghrelin concentration during the day was analyzed by repeated-measures ANOVA with Bonferroni correction (Supplementary Fig. 6). Data are presented as the means ± s.e.m. *P* values <0.05 were considered to be statistically significant.

## Results

### Food intake, locomotor activity and voluntary wheel-running activity under *ad libitum* feeding

Under *ad libitum* feeding, body weight was comparable between both exercise groups (26.1 ± 0.3 g in the WT-Ex and 24.7 ± 0.6 g in the GKO-Ex at 14 weeks of age). The diurnal rhythm of the food intake and locomotor activity in the WT-Ex mice were comparable with those in GKO-Ex mice during either the light or dark periods (Fig. 1A, B and Supplementary Fig. 3A, B, C, D). Both WT-Ex and GKO-Ex mice showed an obviously diurnal rhythm of voluntary wheel-running activity, most of which was observed during the dark period. In either genotype of mice, marked increases of voluntary wheel-running activity were observed at the beginning of dark period concomitant with an increased food intake and locomotor activity in this period (Supplementary Fig. 3E and F). However, the total amount of voluntary exercise activity during dark period was markedly reduced in GKO-Ex mice compared to that in WT-Ex mice (Fig. 2C).

### Food intake, locomotor activity and voluntary wheel-running activity under time-restricted feeding

Under time-restricted feeding, the cumulative food intakes of both WT-Ex-RF and GKO-Ex-RF mice were comparable (Fig. 2A and Supplementary Fig. 4A, B). It is of interest that the volume of food consumed during the restricted access period was almost equivalent to that consumed during 24 h under *ad libitum* feeding (Figs 1A and 2A). Most of the locomotor activity was observed during the restricted feeding hours (Supplementary Fig. 4C and D), and the cumulative locomotor activity during the light and dark periods was comparable in both of the RF groups (Fig. 2B).

In both RF groups, the voluntary wheel-running activity was obviously tuned in to the restricted access period, predominantly the beginning of dark period (Supplementary Fig. 4E and F). Despite the fact that the amount of voluntary wheel-running activity in each RF groups was greater than that observed in the mice with *ad libitum* access to food, the activity of the GKO-Ex-RF mice was still significantly reduced in comparison to that of the WT-Ex-RF mice (Fig. 2C).

The amount of FAA was measured for the 1-hour term before each 2-h restricted access to food during dark period. FAA at the end of the dark period (ZT21-22) was greater than that at the beginning of the dark period (ZT11-12) in the WT-Ex-RF mice, and significantly higher than that in the GKO-Ex-RF mice at ZT21-22 (Supplementary Fig. 5).

### The wheel-running activity patterns with *ad libitum* or time-restricted feeding

When the voluntary wheel-running activity patterns for every 2 h during the dark period were analyzed, cumulative wheel running activity during each 2-h period was markedly greater in the WT-Ex than that in the GKO-Ex, especially at the beginning of dark period (ZT12-14 and 14-16) under *ad libitum* feeding (Fig. 3A). Under time-restricted feeding, dominant increases of wheel-running activity were observed either at the beginning or at the end of dark period in both exercise groups corresponding to the restricted feeding hours. However, there still remained a significant reduction of wheel running activity in the GKO-Ex-RF compared to the WT-Ex-RF at the beginning of dark period (ZT14-16, Fig. 3B).

### The effect of a ghrelin receptor agonist on voluntary wheel running activity in GKO mice

The plasma ghrelin levels in the WT-Ex mice fluctuate during the day with its peak at ZT12, corresponding to the beginning of the dark period, and being higher than those in the WT mice without exercise at every time point except for ZT 6 (Supplementary Fig. 6). These results prompted us to design another experimental protocol to investigate whether an artificial elevation of plasma ghrelin concentration at the beginning of dark period in the GKO mice could reproduce an obvious diurnal rhythm of voluntary exercise which had been observed in the WT-Ex mice.

The effects of ghrelin receptor agonist (GHRP-6) administration on voluntary exercise in GKO mice are shown in Fig. 4. After the once-daily administration of GHRP-6 at the beginning of dark period for 14 days, the wheel running activity was dramatically increased in comparison to the vehicle-injected group, especially during the first half of dark period (ZT12-14, 14-16, 16-18 and 18-20, Fig. 4A). In contrast, the 24-h continuous administration of GHRP-6 by an osmotic mini-pump for 14 days had no effect on the wheel running activity during the dark period in GKO mice (Fig. 4B).

### The relevance of ghrelin on the feeding-induced dopamine levels in the unilateral NAc

The basal dopamine (DA) levels in the NAc were comparable between WT-Ex and GKO-Ex (Fig. 5A). In line with the previous reports, after the consumption of food, DA levels immediately increased to over 150% of the basal levels (Hernandez & Hoebel 1988, Kawahara *et al.* 2009, 2013) in WT-Ex mice and was persistently higher and did not return to the basal levels for up to 140 min after food consumption. Those increases in the DA levels of the GKO-Ex mice were much smaller and significantly lower than those observed in the WT-Ex mice. By the administration of GHRP-6 10 min before feeding, however, the increase of DA was restored to the levels of WT-Ex mice up to 60 min and at 120 min after food consumption (Fig. 5B).

## Discussion

The major outcomes of the present study were as follows. First, voluntary exercise activity was dominantly observed at the beginning of the dark period and was severely attenuated in GKO mice under *ad libitum* feeding. Secondly, under time-restricted feeding, both WT and GKO mice performed a greater amount of voluntary exercise than mice with *ad libitum* access to food; however, the amount of voluntary exercise in the GKO mice was still significantly lower in comparison to WT mice. Thirdly, the plasma ghrelin concentration fluctuates throughout the day with its peak at the beginning of the dark period under voluntary exercise. The single administration of a ghrelin agonist at the beginning of the dark period markedly enhanced the voluntary exercise activity of GKO mice to a level that was comparable to that of WT mice. In contrast, the continuous administration of the same dose of the agonist had no obvious effect. Finally, post-prandial enhancement of dopamine levels in the NAc were significantly attenuated in GKO mice and significantly restored by the administration of GHRP-6 as a ghrelin receptor agonist.

Ghrelin promotes the sensation of hunger and appetite. It is therefore recognized as a food-initiating hormone. Apart from ‘homeostatic hunger’ to maintain an essential energy balance, most people in modern society are predisposed to ‘hedonic hunger’, which is driven by pleasure with the spread of palatable food that is rich in fat and sugar (Lowe & Butryn 2007). Hedonic hunger is recognized as a behavior based on the motivation to eat (Lowe & Butryn 2007), and ghrelin is known to be related to higher motivation to explore for food as FAA (Blum *et al.* 2009, Merkestein *et al.* 2012, Menzies *et al.* 2013). In the present study FAA was observed under restricted feeding condition, which was considerably attenuated in GKO mice. It has been reported that ghrelin directly targeted VTA and increased food motivation as reward-driven behaviors (Skibicka *et al.* 2011), indicating an essential relationship between this peptide and central reward circuit. We previously reported that the induction of voluntary exercise in HFD-induced obese rats drastically ameliorated abnormal feeding behavior together with a concomitant restoration in the production of ghrelin (Mifune *et al.* 2015), suggesting the existence of possible crosstalk between ghrelin and exercise. Considering an essential role of ghrelin in the reward circuit, it is further speculated that ghrelin is relevant to the motivation to perform exercise apart from feeding. We hypothesized that ghrelin plays a crucial role as an initiator of voluntary exercise. To test this issue, ghrelin-knockout mice were used for the measurement of voluntary exercise activity.

With *ad libitum* access to food, the intake of food and locomotor activity were mainly observed during the dark period. These behavioral activities were most frequently observed at the beginning of the dark period, followed by the end of this period. The voluntary wheel running activity was concentrated in the same period as that in which the intake of food and locomotor activity was frequently observed, suggesting an integral relationship between feeding and exercise as similar motivational activities. There were no differences of food intake and locomotor activity between in the GKO and WT mice. Taking the nature of ghrelin as an appetite-promoting hormone into account, it is paradoxical that deleting ghrelin had no apparent effect on cumulative food intake compared to that in the WT mice. In this context, some former reports demonstrated that ablation of ghrelin did not affect food intake in either obese (Sun *et al.* 2006) or lean model (Sato *et al.* 2008), suggesting the possibility that some compensating mechanisms may work to regulate homeostatic feeding behavior. In contrast, the voluntary exercise of GKO mice was conspicuously reduced in comparison to WT mice. It is therefore suggested that there may be an underlying relationship between ghrelin and voluntary exercise, especially at the beginning of dark period.

Under time-restricted feeding, in which rodents have limited access to food at a fixed time point of their circadian phase, an increase in locomotor activity and voluntary exercise occurs as an exploratory behavior (Hatori *et al.* 2012). As expected, the amount of voluntary exercise in both the WT and GKO mice was greater than that observed in mice with *ad libitum* access to food; however, in GKO mice, the amount was still significantly attenuated in comparison to WT mice. It is therefore plausible that the difference of voluntary exercise between those two groups with different genotype is attributable to the lack of ghrelin, suggesting an essential role of this hormone as a promoter of exercise.

In the present study, we demonstrated for the first time that the plasma ghrelin concentration fluctuates throughout the day with its peak at ZT12, corresponding to the beginning of the dark period. As mentioned above, it is likely that there is a possible relationship between exercise and appetite-regulating hormones. Indeed, a marked increase in the plasma ghrelin concentration was observed at this time point in the exercise group. The fact that the voluntary wheel running activities were markedly enhanced at this time point suggests that the peak of ghrelin production at a fixed time in their circadian rhythmicity is essential for the initiation of voluntary exercise. Under both *ad libitum* and time-restricted feeding conditions, persistent voluntary exercise was mainly observed at the beginning of the dark period and became shorter and temporarily increased just before the end of dark period. These findings prompted us to perform further experiments to determine whether the exogenous administration of a ghrelin agonist to GKO mice could reproduce the rhythm of voluntary exercise, which had been observed in WT mice. The single administration of GHRP to GKO mice at the beginning of dark period led to a marked increase in the amount of voluntary exercise – especially during the first half of this period. In contrast, the continuous administration of this agent had no effect at all. These results strongly support our hypothesis that ghrelin is an essential initiator of voluntary exercise as well as feeding behavior, and that a dynamic surge of the plasma ghrelin concentration, rather than the persistent concentration of this peptide is critical for the initiation of voluntary exercise during this period.

The post-prandial dopamine levels in the NAc were markedly enhanced after food intake under voluntary exercise in the WT mice in contrast to the considerably smaller increment in the GKO mice. Because the high expression of GHSR is recognized in dopamine neurons in the VTA (Zigman *et al.* 2006), which project to the NAc, these dopamine neurons in the VTA represent a critical site for ghrelin to trigger food consumption as a motivational behavior. It is well known that ghrelin is associated with the brain reward pathway, including the intake of food and food preferences (Menzies *et al.* 2013). Locomotion is an important aspect of feeding-related behavior. An animal must forage or hunt for food before eating. Ghrelin increases the locomotor activity in mice when it is administered centrally into the third ventricle (Jerlhag *et al.* 2006), which suggests a possible role of this peptide as an initiator of motivational behavior through the brain reward circuit. In the present study, the voluntary wheel running activity of GKO mice was disturbed rather than their locomotor activity. Taken together with the lower dopamine levels in the NAc of GKO mice, it is conceivable that motivational reward systems are involved in the mechanisms of ghrelin’s effects as an initiator of voluntary exercise.

In the present study, it is noteworthy that a peripherally produced molecule such as ghrelin was transmitted to the CNS and that it affected the reward circuit via the dopamine system. Although the central administration of ghrelin directly to the VTA has been shown to be associated with the release of dopamine and motivational feeding (Abizaid *et al.* 2006, Jerlhag *et al.* 2007, Cone *et al.* 2015), few reports have demonstrated that the systemic administration of ghrelin can affect the dopamine neurons in the VTA (Kawahara *et al.* 2009, 2013). The production of ghrelin in the stomach was accelerated at the beginning of the dark period due to some unknown mechanisms related to hunger, feeding or the lights being turned off during this period. It is assumed that an enhanced ghrelin signal was transmitted to the central mesolimbic area and activated the reward circuit, which then motivated voluntary exercise. This relationship between ghrelin and exercise motivation through a central reward circuit is a novel finding of the present study.

In conclusion, it is plausible that a surge in ghrelin at the beginning of the dark period, which was clearly observed in WT mice, plays a crucial role in the initiation and motivation of voluntary exercise related to the central dopamine system. Results obtained from the present study may offer a proposal for new strategies to perform or adhere to exercise in terms of health and fitness, holding ghrelin as motivational properties in great account. In the present study, however, dopamine concentrations in each exercise group of mice were measured after the termination of exercise protocol, and those during real-time exercise performance have not been clarified yet. To this end, more special system should be developed to measure both exercise amount and dopaminergic activity simultaneously in the future mechanistic studies.

## Supplementary Material

Supplementary Figure 1 Side (A) and top (B) views of a specially designed polyvinyl chloride chamber. This special chamber (W32×D20.5×H26.5 cm) was equipped with a running wheel apparatus (15 cm diameter and 5 cm width) for recording the food intake and locomotor activity as well as the wheel-running activity in an automatically monitored system. SR: Sedentary room, ER: Exercise room, RW: Running wheel.

Supplementary Figure 2 Representative location of a microdialysis probe placed in the mouse NAc.

Supplementary Figure 3 Representative results of food intake (a, b), locomotor activity (c, d) and wheel-running counts (e, f) under ad libitum feeding during the light and the dark periods in the WT-Ex mice (a, c, e) and the GKO-Ex mice (b, d, f).

Supplementary Figure 4 Representative results of food intake (a, b), locomotor activity (c, d) and wheel-running counts (e, f) under time-restricted feeding (RF) during the light and the dark periods in the WT-Ex mice (a, c, e) and the GKO-Ex mice (b, d, f).

Supplementary Figure 5 The food anticipatory locomotor activity under time-restricted feeding.

Supplementary Figure 6 The plasma concentration of ghrelin in the sedentary and exercise groups. The plasma ghrelin levels in both WT and WT-Ex mice during the light and the dark periods. Values are means±S.E.M. WT, n=9; WT-Ex, n=10. *P<0.05, ***P<0.001 versus WT at the same time point. †P<0.01 vs. WT-Ex at ZT 0, ZT 6 or ZT 18.

## Declaration of interest

The authors declare that there is no conflict of interest that could be perceived as prejudicing the impartiality of the research reported.

## Funding

This work was supported in part by Grant-in-Aid for Scientific Research (C) (No. 25504019 and 25350914) from the Ministry of Education, Culture, Sports, Science and Technology of Japan. This work was further supported by the Projects of Fukuoka Prefectural Bio Industry Center Promotion Conference through Kurume Research Park Co., Ltd.

## Author contribution statement

H M, Y T, A N and M K participated in the planning and designing the experiments. H M, Y T, Y S, T S, Y K and K H performed the animal experiments. R M helped with animal experiments. Y N performed the measurement of plasma ghrelin concentration. Y T and T K performed statistical analysis. M K created ghrelin-knockout mice. H M and Y T wrote the manuscript. All authors reviewed and commented on the manuscript.
