# Supplementary figures and images for: Voluntary exercise is motivated by ghrelin, possibly related to the central reward circuit
Source: J Endocrinol. 2019 Oct 8;244(1):123–32. doi: 10.1530/JOE-19-0213 (PMC6859445; doi:10.1530/JOE-19-0213)

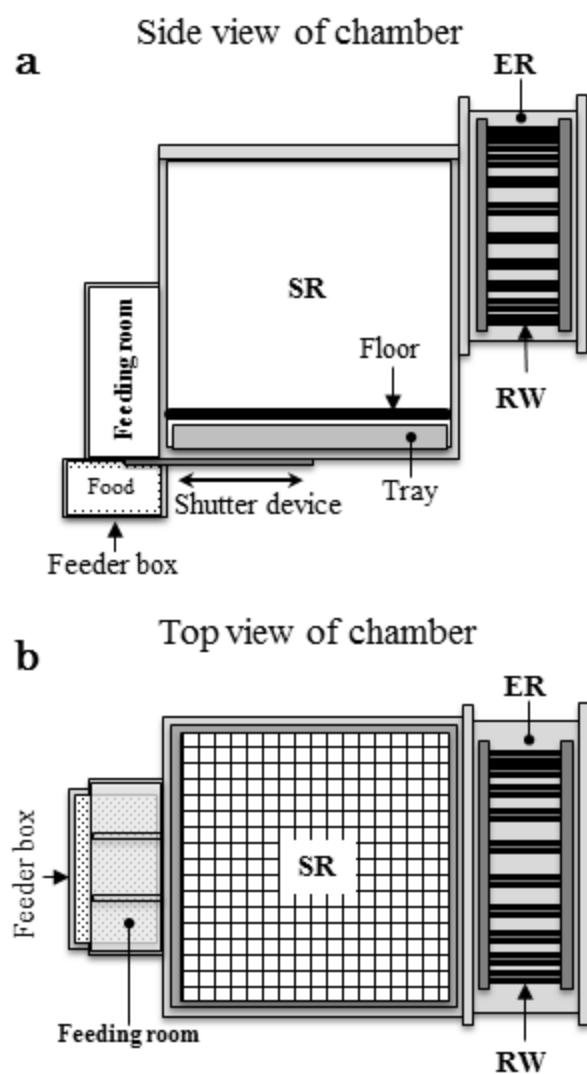

**Supplementary Figure 1** Mifune H *et.al.*

Supplement: Supplementary Figure 1 Side (A) and top (B) views of a specially designed polyvinyl chloride chamber. This special chamber (W32×D20.5×H26.5 cm) was equipped with a running wheel apparatus (15 cm diameter and 5 cm width) for recording the food intake and locomotor activity as well as the wheel-runnin [file supplementary_figure_1.pdf]

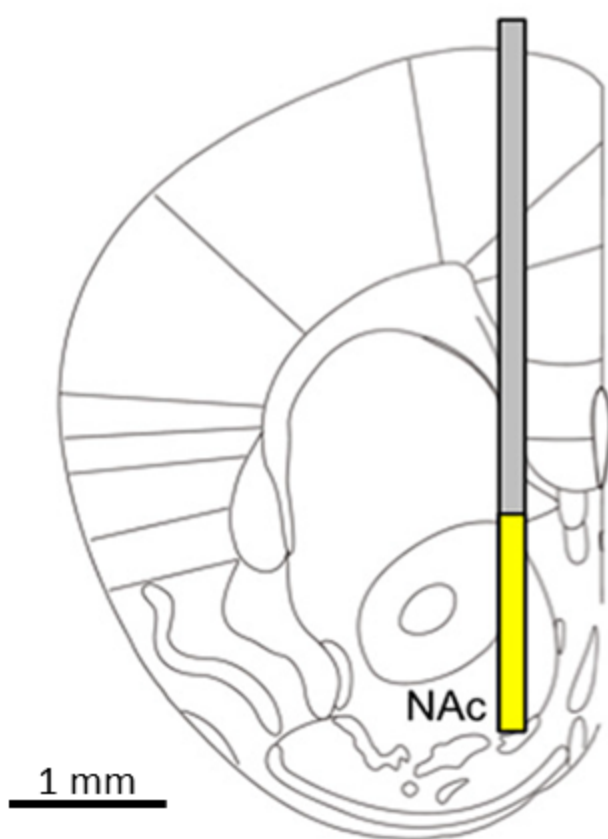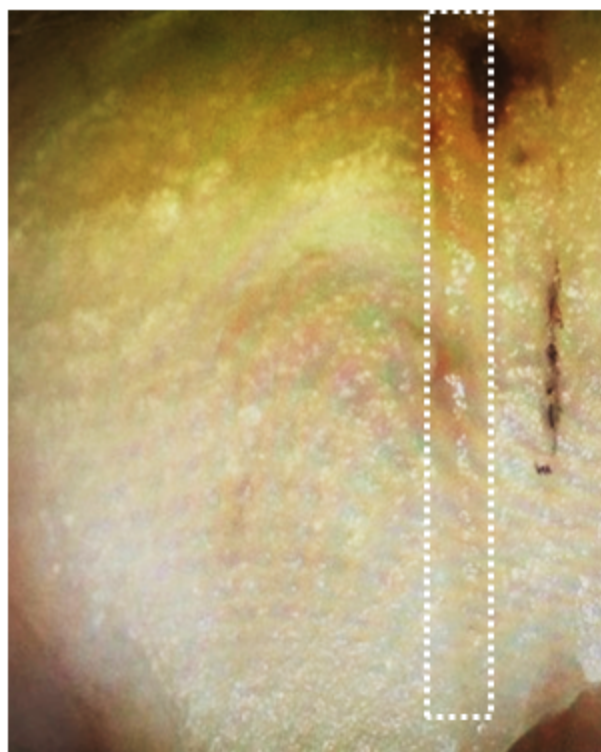

**Supplementary Figure 2** Mifune H *et.al.*

Supplement: Supplementary Figure 2 Representative location of a microdialysis probe placed in the mouse NAc. [file supplementary_figure_2.pdf]

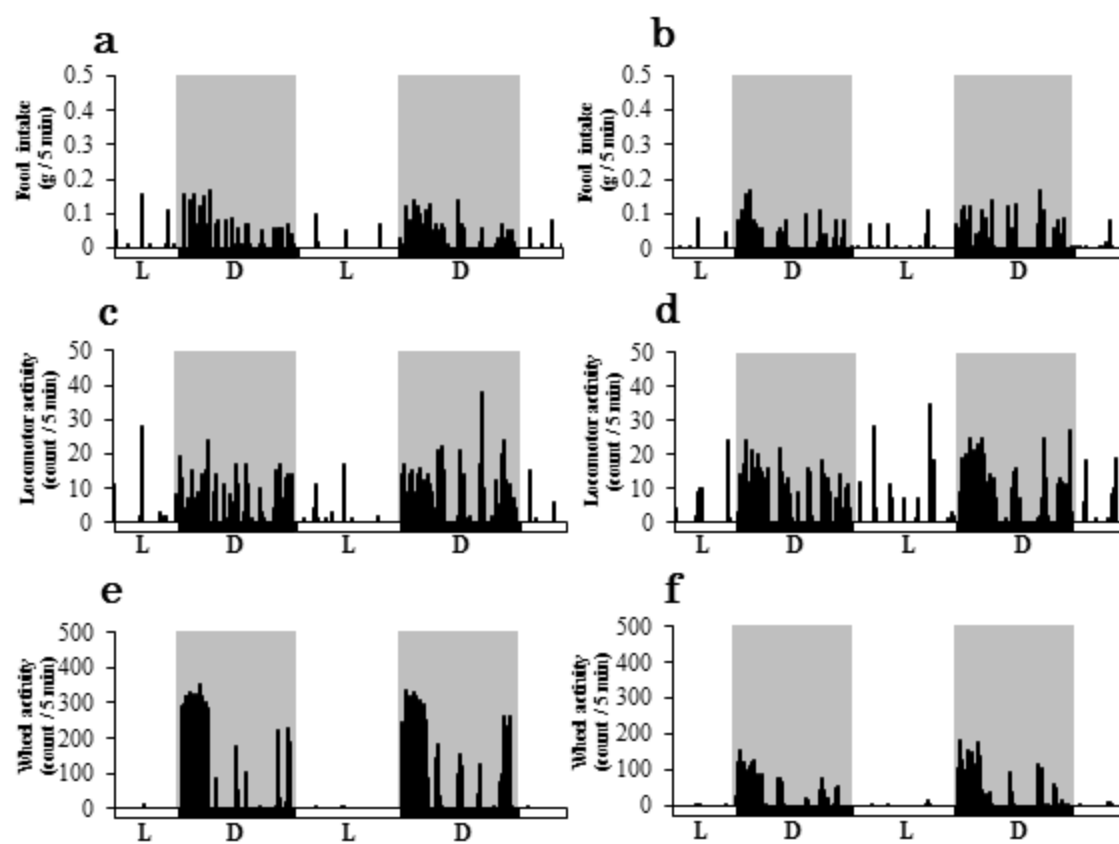

**Supplementary Figure 3** Mifune H *et.al.*

Supplement: Supplementary Figure 3 Representative results of food intake (a, b), locomotor activity (c, d) and wheel-running counts (e, f) under ad libitum feeding during the light and the dark periods in the WT-Ex mice (a, c, e) and the GKO-Ex mice (b, d, f). [file supplementary_figure_3.pdf]

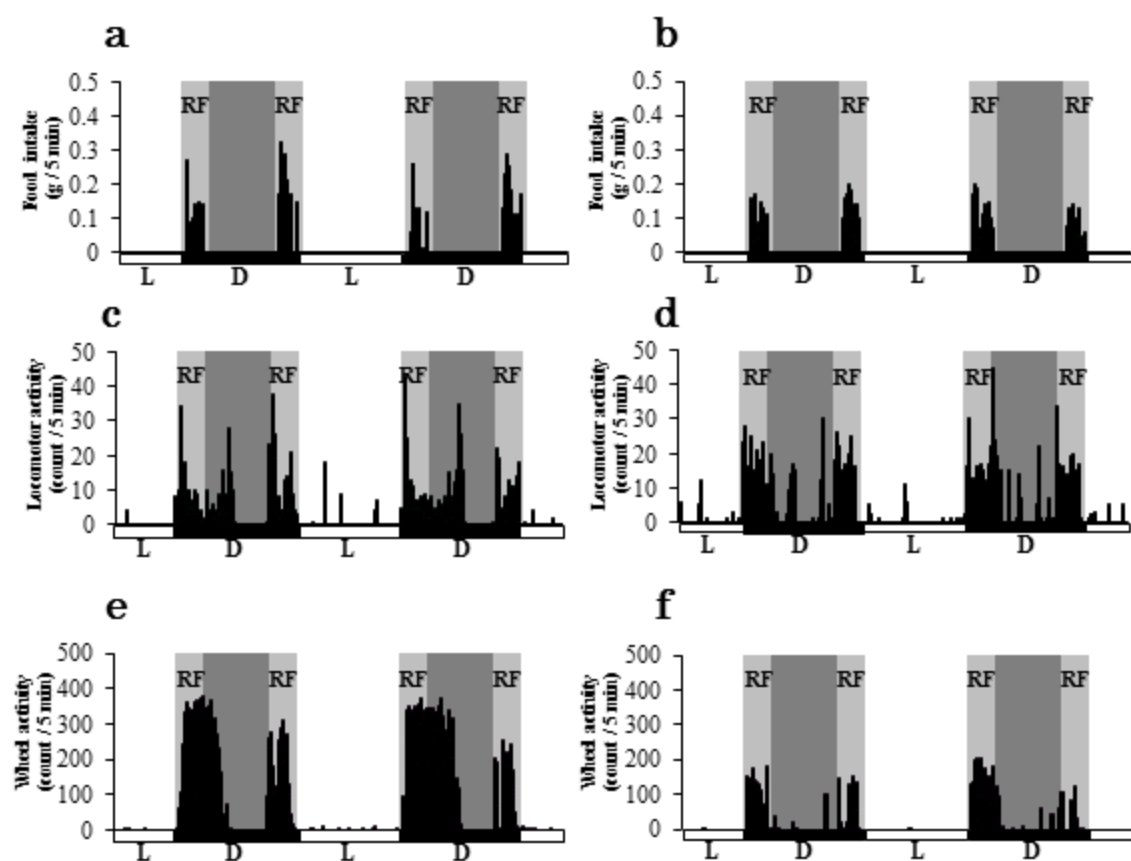

**Supplementary Figure 4** Mifune H *et.al.*

Supplement: Supplementary Figure 4 Representative results of food intake (a, b), locomotor activity (c, d) and wheel-running counts (e, f) under time-restricted feeding (RF) during the light and the dark periods in the WT-Ex mice (a, c, e) and the GKO-Ex mice (b, d, f). [file supplementary_figure_4.pdf]

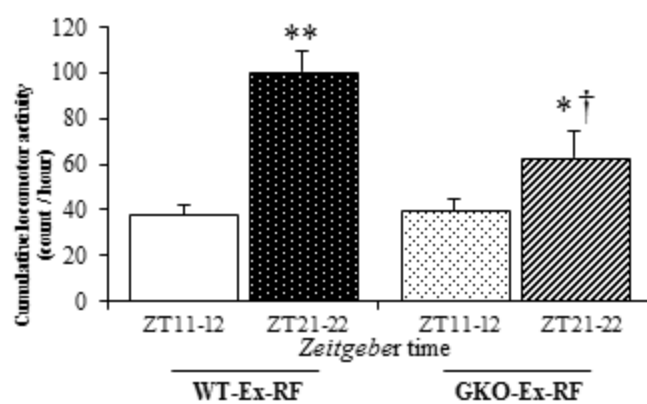

**Supplementary Figure 5** Mifune H *et.al.*

Supplement: Supplementary Figure 5 The food anticipatory locomotor activity under time-restricted feeding. [file supplementary_figure_5.pdf]

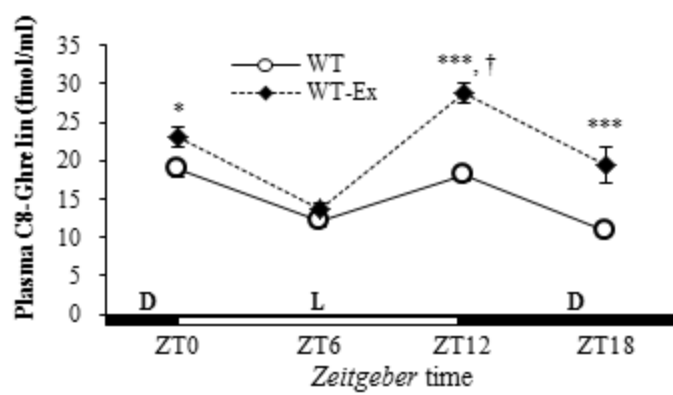

**Supplementary Figure 6** Mifune H *et.al.*

Supplement: Supplementary Figure 6 The plasma concentration of ghrelin in the sedentary and exercise groups. The plasma ghrelin levels in both WT and WT-Ex mice during the light and the dark periods. Values are means±S.E.M. WT, n=9; WT-Ex, n=10. *P<0.05, ***P<0.001 versus WT at the same time point. †P<0.01 vs.  [file supplementary_figure_6.pdf]
